# A study on the combination of functional connection features and Riemannian manifold in EEG emotion recognition

**DOI:** 10.3389/fnins.2023.1345770

**Published:** 2024-01-15

**Authors:** Minchao Wu, Rui Ouyang, Chang Zhou, Zitong Sun, Fan Li, Ping Li

**Affiliations:** ^1^Anhui Province Key Laboratory of Multimodal Cognitive Computation, School of Computer Science and Technology, Anhui University, Hefei, China; ^2^Key Laboratory of Flight Techniques and Flight Safety, Civil Aviation Flight University of China, Guanghan, China

**Keywords:** emotion recognition, human-computer interface (HCI), electroencephalogram (EEG), functional connection feature, Riemannian manifold, decision fusion

## Abstract

**Introduction:**

Affective computing is the core for Human-computer interface (HCI) to be more intelligent, where electroencephalogram (EEG) based emotion recognition is one of the primary research orientations. Besides, in the field of brain-computer interface, Riemannian manifold is a highly robust and effective method. However, the symmetric positive definiteness (SPD) of the features limits its application.

**Methods:**

In the present work, we introduced the Laplace matrix to transform the functional connection features, i.e., phase locking value (PLV), Pearson correlation coefficient (PCC), spectral coherent (COH), and mutual information (MI), to into semi-positive, and the *max* operator to ensure the transformed feature be positive. Then the SPD network is employed to extract the deep spatial information and a fully connected layer is employed to validate the effectiveness of the extracted features. Particularly, the decision layer fusion strategy is utilized to achieve more accurate and stable recognition results, and the differences of classification performance of different feature combinations are studied. What's more, the optimal threshold value applied to the functional connection feature is also studied.

**Results:**

The public emotional dataset, SEED, is adopted to test the proposed method with subject dependent cross-validation strategy. The result of average accuracies for the four features indicate that PCC outperform others three features. The proposed model achieve best accuracy of 91.05% for the fusion of PLV, PCC, and COH, followed by the fusion of all four features with the accuracy of 90.16%.

**Discussion:**

The experimental results demonstrate that the optimal thresholds for the four functional connection features always kept relatively stable within a fixed interval. In conclusion, the experimental results demonstrated the effectiveness of the proposed method.

## 1 Introduction

Affective computing is a science involving multiple disciplines, such as psychology, biology, and philosophy, among which research on affective models and emotion recognition are two important branches in the field of affective computing. Generally, affective models can be mainly categorized as discrete models and dimensional models. Discrete emotion theorists think that people's emotions consist of several basic emotions, such as Ekman's (1992) six-basic emotions model and Izard's (2007) 10-basic emotions model. However, dimensional emotion theorists believe that human's emotions are continuous and have some special characteristics, such as the valence-arousal model (Russell, 1980) and the Plutchik (2001)'s Wheel of Emotions.

With the development of information technology and artificial intelligence technology, emotion recognition plays an increasingly important role in the field of human–computer interaction, because machines can become more intelligent through emotional interaction with humans (Picard, 2003). For this reason, emotion recognition has received widespread attention from researchers in various fields, such as emotion recognition based on facial expressions, emotion recognition based on speech, and emotion recognition based on EEG signals (Koolagudi and Rao, 2012; Ko, 2018; Abramson et al., 2020; Houssein et al., 2022; Yi et al., 2024). However, among the many directions of emotion recognition, EEG signals can establish a closer and more realistic mapping relationship with emotions due to their “unconcealability,” that is to say, people can easily disguise their true emotions by adjusting their expressions and voices, but it is difficult to change the corresponding EEG signals at the same time.

Feature extraction is one of the core modules in the EEG emotion recognition process for highly discriminative features that can help improve the performance of emotion recognition models. In recent years, various features have been developed to solve the EEG-based emotion recognition task, where these features can been roughly divided into two classes, i.e., the single-channel features and the multi-channel features. The calculation of single-channel features does not depend on other channels of EEG, therefore, the features of each channel can be considered independent of each other. Duan et al. (2013) first developed differential entropy (DE) to decode the EEG signals and classify different emotional states, then Zheng and Lu (2015) verified that the DE features have higher discrimination and robustness compared with other features by experiments in two public emotional datasets, i.e., the SEED and the DEAP (Koelstra et al., 2011). Moreover, the sample entropy (SE) and the approximate entropy (ApEn) are also two common entropy-based features to measure the uncertainty of emotional EEG signals (Zeng et al., 2019; Wang et al., 2022). In addition, the wavelet energy and the power spectral density (PSD) are also two common features to measure the frequency domain information for EEG signals (Zheng and Lu, 2015; Mohammadi et al., 2017; Wang et al., 2022). Compared with single-channel features, multi-channel features, or functional connection features, are more concerned with measuring the interactive information between channels. The differential asymmetry (DASM) and the rational asymmetry (RASM) are widely employed to measure the difference of EEG signals between the left and right hemispheres (Zheng and Lu, 2015; Zhang et al., 2021). Both Dasdemir et al. (2017) and Nguyen and Artemiadis (2018) used the phase-locking value (PLV) to build a functional brain neural network and perform the emotion recognition task. Khosrowabadi et al. (2010) utilized the mutual information (MI) to establish a dynamic emotional system. In addition, the Pearson correlation coefficient (PCC), the spectral coherence coefficient (COH), the phase lag index (PLI), and the covariance matrix (COV) are also usually adopted to represent the emotional interaction information between different channels of EEG (Jadhav et al., 2017; Keelawat et al., 2021; Wu et al., 2022; Lin et al., 2023).

Furthermore, a large number of techniques have been developed to construct the mapping relationship between EEG features and emotions. Zheng and Lu (2015) introduced the deep belief network (DBN) to decoding the DE features and achieved 86.08% accuracy for positive, negative, and neutral states. Du et al. (2020) proposed an attention-based LSTM with Domain Discriminator (ATDD-LSTM) with DE as input features to solve the subject-dependent and subject-independent emotion recognition tasks in three public emotional datasets. Moon et al. (2020) employed the convolutional neural networks (CNNs) to extract the spatial domain information from PLV and PCC and transform entropy (TE). Song et al. (2018) developed a novel dynamical graph convolutional neural networks (DGCNNs) to model the multichannel EEG features and perform the emotion recognition task and achieved average accuracies of 90.4% and 79.95% for subject dependent and subject independent cross-validation on the SEED, respectively. Liu et al. (2023) combined the attention mechanism and pre-trianed convolutional capsule network to extract the spatial information from the original emotional EEG signals. Zali-Vargahan et al. (2023) introduced CNN to extract the deep time-frequency features and employed several machine classifiers such as decision tree to classify different emotional states, where the average accuracy of 94.58% had been achieved in SEED. Ma et al. (2023) developed the transfer learning methods to reduce the distribution differences of emotional EEG signals between different subjects, thereby enabling more robust cross-subject emotion recognition.

In recent years, Riemannian manifolds (RMs) have received a lot of attention in the field of brain–computer interfaces due to the simplicity, accuracy, robustness, and transfer learning capabilities (Congedo et al., 2017). Barachant et al. (2013) developed the Riemannian-based kernel support vector machine (RK-SVM) to solve the motor image task in a public brain–computer interface (BCI) competition dataset. However, since the conventional deep learning model mainly utilize the non-linear function to map the features located in the Euclidean space, the features located in RM usually cannot be fed into the deep model, for the non-linear function will change the SPD of the features and the mapped features will then locate in Euclidean space. Therefore, Huang and Van Gool (2017) designed a RM network architecture by the combination of bilinear and non-linear learning, and achieved the state-of-the-art accuracies in three datasets. Yair et al. (2019) utilized the parallel transport to achieve the domain adaptation for symmetric positive definite (SPD) matrices in RM, and achieve accuracy of 78% for four-class motor imagery task by the leave-one-session-out cross-validation. In addition, it can also map Riemannian features to Euclidean space by establishing a mapping between Riemannian manifold and Euclidean space, and then use deep learning methods (Wu et al., 2022). Particularly, in the domain of affective BCIs, Riemannian manifolds approaches have been instrumental in feature extraction and classification tasks related to emotion recognition. The utilization of covariance matrices and manifold-based representations allows for a more nuanced understanding of the underlying neural patterns associated with different emotional states (Abdel-Ghaffar and Daoudi, 2020; Wang et al., 2021). However, the limitation of the Riemannian manifold is that its features must be symmetric and positive definite, which greatly limits the application of the Riemannian methods.

In the present study, we adopted four functional connection features, i.e., PLV, PCC, COH, and MI, to perform the emotion recognition task in the SEED database. The main contributions of this study can be summarized as follows: (1) We introduced the the Laplace matrix with a *max* operation to transform the four functional connection features into SPD; (2) Four functional connection features almost achieved similar performances in recognizing emotions, especially PLV, PCC, and COH, which may indicate the stability of brain functional connection when subjects are evoked emotions; (3) The decision fusion strategy is employed to fuse the four features to achieve higher accuracy and robustness, and a detailed comparison about the combination for different features in emotion recognition has been made.

The layout of this study is organized into following sections: In Section 2, the detailed descriptions about the four functional connection features, the SPDnet, the Laplace matrix, and the overview about the proposed model are presented. The detailed experimental results, analysis, and discussion for the SEED dataset are presented in Section 3. Finally, Section 4 presents the conclusion about this study, as well as the discussion about future works.

## 2 Materials and methods

### 2.1 Feature extraction

In this study, four functional connection features are adopted to measure the effectiveness of the proposed method and recognize emotional states. Specially, denote the $X_{i}\in {\mathbb{R}}^{T}$, *i* = 1, …, *C* as the *i*-th channel EEG signals where *C* and *T* represent the number of EEG channel and the time length, respectively. Table 1 displays all of main variables and the corresponding meaning.

**Table 1:** The parameter details of the proposed model.

**Variable**	**Meaning**	**Variable**	**Meaning**
ℝ	Real number field	*X*	EEG signals
M	Riemannian manifold	*Sym* ^+^	SPD matrices set
$T_{S}M$	Tangent Space of *S*	*S*_*i*_	SPD matrix
*T*_*i*_	Point in $T_{S}M$	ϵ	Threshold value
*U*	Eigenvector matrix	*V*	Diagonal matrix of eigenvalue
*I*	Identity matrix	*W*	Transformation matrix in SPDnet
*A*	Undirected adjacency matrix	*C*	Number of EEG channels
*D*	Degree matrix	*L*	Laplace matrix

#### 2.1.1 Phase locking value

The PLV is a phase-based method, which measures the phase difference between the two channel signals (Gysels and Celka, 2004). The calculation formula of PLV is defined as Equation (1),


(1) $$PLV_{m,n}=|\frac{1}{T}\sum_{t=1}^{T}e^{j(\phi_{m,t}-\phi_{n,t})}|$$

Where ϕ_*m, t*_ and ϕ_*n, t*_ represent the phase angles of signals *X*_*m*_ and *X*_*n*_, respectively, at time point *t*.

#### 2.1.2 Pearson correlation coefficient

The PCC measures the linear correlation degree between two channel signals in time domain (Guevara and Corsi-Cabrera, 1996). The value range of PCC is [–1,1]; hence, PCC can measure whether two signals are positively or negatively correlated. The calculation formula of PCC is defined as Equation (2),


(2) $$PCC_{m,n}=\frac{\sum_{t=1}^{T}\left(X_{m,t}-{\bar{X}}_{m})(X_{n,t}-{\bar{X}}_{n}\right)}{\sqrt{\sum_{t=1}^{T}{\left(X_{m,t}-{\bar{X}}_{m}\right)}^{2}}\sqrt{\sum_{t=1}^{T}{\left(X_{n,t}-{\bar{X}}_{n}\right)}^{2}}}$$

Where ${\bar{X}}_{m}$ and ${\bar{X}}_{n}$ are expectations of *X*_*m*_ and *X*_*n*_, respectively.

#### 2.1.3 Spectral coherence

Contrary to PCC, the COH measures the linear correlation degree between two channel signals in frequency domain (Guevara and Corsi-Cabrera, 1996). The calculation formula is defined as Equation (3),


(3) $$\begin{array}{l} Coh_{m,n}=\frac{|P_{m,n}\left(f\right){|}^{2}}{P_{m,m}\left(f\right)P_{n,n}\left(f\right)} \end{array}$$

Where *P*_*m, n*_(*f*) means the cross-spectral density of EEG signal *X*_*m*_ and *X*_*n*_ in the frequency *f*, while *P*_*m, m*_ and *P*_*n, n*_ means the power spectral density of *X*_*m*_ and *X*_*n*_, respectively.

#### 2.1.4 Mutual information

MI is a information theory based method where advantage of MI is that it can detect the linear and non-linear correlation of two signals at the same time (Jeong et al., 2001). However, the accuracy of MI calculation is easily affected by the noise in the signal and the length of the signal. The calculation formula is defined as Equation (4),


(4) $$\begin{array}{l} MI_{m,n}=\underset{X_{m},X_{n}}{\sum }P_{X_{m},X_{n}}\left(x_{m},x_{n}\right)\log_{2}\frac{P_{X_{m},X_{n}}\left(x_{m},x_{n}\right)}{P_{X_{m}}\left(x_{m}\right)P_{X_{n}}\left(x_{n}\right)} \end{array}$$

Where *P*_*X*_*m*_, *X*_*n*__ means the joint probability distribution of *X*_*m*_ and *X*_*n*_, and *P*_*X*_*m*__ and *P*_*X*_*n*__ mean the probability distributions of *X*_*m*_ and *X*_*n*_, respectively.

### 2.2 SPD metrix network

#### 2.2.1 Riemainnian manifold

The SPD set is defined as Equation (5) as following


(5) $$\begin{array}{l} Sym^{+}=S\in {\mathbb{R}}^{C\times C},x^{⊤}Sx>0,S=S^{⊤},\forall x\in {\mathbb{R}}^{C} \end{array}$$

and lie on the Riemannian manifold (RM) M rather than the Euclid space. However, There are two operators that can realize the mutual mapping between Riemannian manifold and Euclidean space. Concretely, suppose there is a point *S*∈*Sym*^+^, then the tangent space of *S*, denoted as $T_{S}M$, could be defined and belongs to the Euclid space. The *logarithmicmap* operator as following is defined to project the point $S_{i}\in Sym^{+}$ to the $T_{S}M$ shown in Equation (6),


(6) $$\begin{array}{l} T_{i}=Log_{S}\left(S_{i}\right)=S^{1/2}logm\left(S^{-1/2}S_{i}S^{-1/2}\right)S^{1/2} \end{array}$$

where *logm* denotes the logarithm of a matrix calculated as Equation (7),


(7) $$\begin{array}{l} logm\left(S\right)=Ulog\left(V\right)U^{-1} \end{array}$$

where *U* = [_*u*_*i*_*j*]*C*×*C*_ and *V* = *diag*{*v*_1_, *v*_2_, …, *v*_*C*_} are the eigenvector and eigenvalue of the matrix *S*. Moreover, a corresponding inverse operation, i.e., *exponentialmap*, is also defined to project the points in the $T_{S}M$ to the M shown in Equation (8),


(8) $$\begin{array}{l} S_{i}=Exp_{S}\left(T_{i}\right)=S^{1/2}expm\left(S^{-1/2}T_{i}S^{-1/2}\right)S^{1/2} \end{array}$$

where *expm* denotes the exponential of a matrix calculated as Equation (9),


(9) $$\begin{array}{l} expm\left(S\right)=Uexp\left(V\right)U^{-1} \end{array}$$

**Figure 1:**
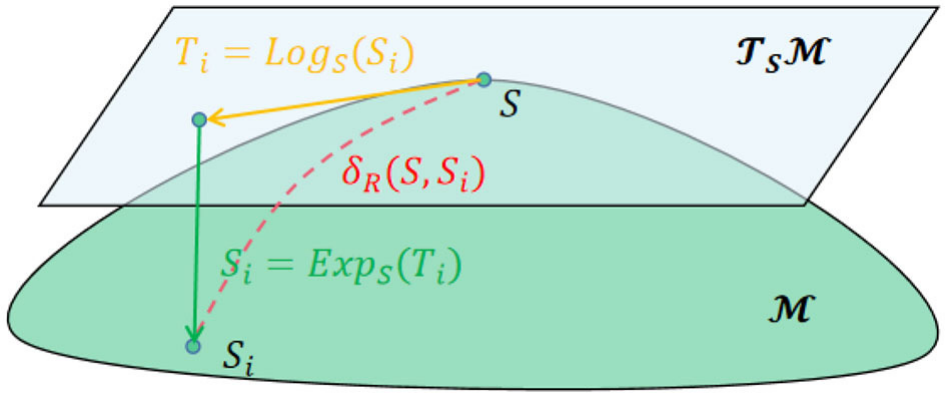
The two operations between the RM and corresponding tangent space in *S*. Particularly, δ_*R*_ represents the geodesic distance between *S* and *S*_*i*_ where the calculation method can be found in Barachant et al. (2013).

Figure 1 shows the two operations between the RM and corresponding tangent space in *S*.

#### 2.2.2 SPDnet

**Figure 2:**
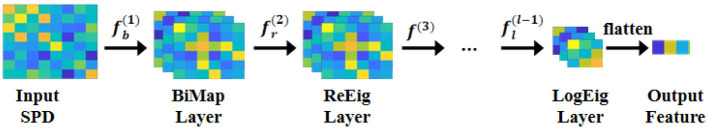
A sample architecture of the SPDnet.

The SPD matrix network (SPDnet), as introduced by Huang and Van Gool (2017), operates analogously to the commonly employed convolutional network (ConvNet). It effectively preserves the inherent geometric information of the SPD matrix, akin to the way ConvNets capture spatial features in other types of data. The SPD net mainly consists of three kinds of layers, i.e., the bilinear mapping (BiMap) layer, the eigenvalue rectification (ReEig) layer, and the log eigenvalue (LogEig) layer, where the BiMap layer is designed to transform the SPD set into a new SPD set by a bilinear mapping, the ReEig layer is designed to rectify the new SPD matrices by a non-linear function to ensure the positive definite of the new SPD matrices, the LogEig layer is designed to perform corresponding RM computing on the output new SPD matrices. Particularly, let *S*_*k*−1_ be the input SPD matrix and the *S*_*k*_ be the output, then the calculation of the three layer can be defined as Equations (10)–(12),


(10) $$\begin{array}{l} S_{k}=f_{b}^{k}\left(S_{k-1};W_{k}\right)=W_{k}S_{k-1}W_{k}^{⊤} \end{array}$$

(11) $$\begin{array}{l} S_{k}=f_{r}^{k}\left(S_{k-1}\right)=U_{k-1}\max\left(\epsilon I,V_{k-1}\right)U_{k-1}^{⊤} \end{array}$$

(12) $$\begin{array}{l} S_{k}=f_{l}^{k}\left(S_{k-1}\right)=log\left(S_{k-1}\right)=U_{k-1}\log\left(V_{k-1}\right)U_{k-1}^{⊤} \end{array}$$

where $f_{b}^{k}$, $f_{r}^{k}$, and $f_{s}^{k}$ are the *k*-th BiMap layer, the *k*-th ReEig layer, and the *k*-th LogEig layer, respectively. In addition, $W_{k}\in {\mathbb{R}}^{d_{k}\times d_{k-1}}$ is the transformation matrix, the *U*_*k*−1_ and the *V*_*k*−1_ are calculated by eigenvalue decomposition (EIG), the ϵ is a rectification threshold, and the *I* is an identity matrix. Figure 2 shows a sample architecture of the SPDnet.

### 2.3 Laplace matrix

Laplace matrix has been widely employed to build the brain functional connection network in the brain–computer interface field, and achieved good performance. Generally, the Laplace matrix is a fundamental concept in graph theory and linear algebra and provides valuable insights into the connectivity and structural properties of a graph's adjacency matrix. Let *A* be an undirected adjacency matrix with *C* nodes, and *D* be the corresponding degree matrix, then the *i*-th element of *D* can be calculated as Equation (13),


(13) $$\begin{array}{l} d_{i}=\overset{C}{\underset{j=1}{\sum }}A_{ij} \end{array}$$

Then, the laplacian matrix *L* is defined as Equation (14),


(14) $$\begin{array}{l} L=D-A \end{array}$$

Typically, the Laplace matrix has an important property, i.e., the Laplace matrix is symmetric, positive, and semi-definite. However, since the Laplace matrix is only positive semi-definite and does not fully satisfy the conditions of the Riemannian manifold, inspired by the ReEig layer in the SPD net, we also introduced the *max* operator to transform the Laplace matrix into positive definite shown in Equation (15) i.e.,


(15) $$\begin{array}{l} L=Umax\left(\epsilon I,\Sigma \right)U^{⊤} \end{array}$$

Therefore, after the *max* operator, the Laplace matrix lies on the RM, and the matrix can be fed into the SPDnet.

### 2.4 Framework design

**Figure 3:**
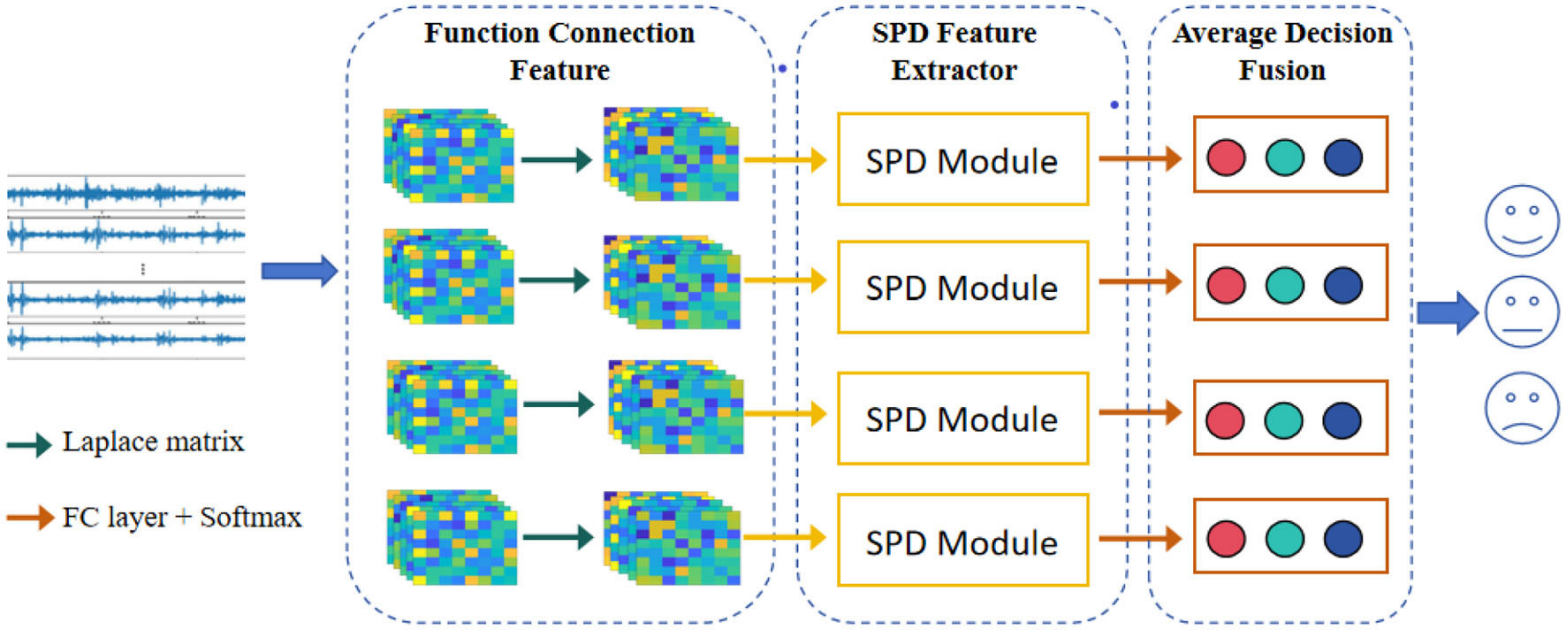
The flowchart of the proposed model. The model mainly consists of three modules: the function connection feature, the deep SPD feature extractor, and the average decision fusion module.

The overview of our model is displayed in Figure 3. As shown in the figure, the model mainly consists of three modules: the function connection feature, the deep SPD feature extractor, and the average decision fusion module. More concretely, in the function connection feature module, four types of feature (i.e., PLV, PCC, MI, and COH) are first extracted from the preprocessed EEG signals, and a threshold value is used to transform the functional connection features into undirected graphs, then the Laplace matrices with the *max* operator are calculated; in the SPD feature extractor module, four BiMap layers, four ReEig layers, and a LogEig layer are combined to further extract the deep features from the Laplace matrices, and the output features are flattened as a vector; in the average decision fusion, considering the difference in information carried by different functional connection features, we used average decision-making layer fusion to synthesize different feature information. The implementation details of the parameters of the proposed model are presented in Table 2.

**Table 2:** The parameter details of the proposed model.

**Parameters**	**Values**
Number of BiMap layer	4
Number of ReEig layer	4
Number of LogEig layer	1
Dimension of each BiMap layer	[31, 20, 16, 12]
Proportion of dropout	0.2
Learning rate	0.001
Batch size	64
Max epochs	200
Weight decay	0.0001

## 3 Experimental results and discussion

### 3.1 Dataset

In this study, the SEED dataset is employed to evaluate the effectiveness of the our method. The SEED dataset is a publicly available emotional dataset and widely used in emotion recognition. A total of 15 healthy subjects (7 male and 8 female participants, mean: 23.27, std: 2.37), who were university or graduate students, were invited to watch 15 film clips with different emotional labels, i.e., positive, negative, and neutral emotional states. That is to say, each experiment contains 15 trials. Particularly, each subject participated in the experiment three times, with at least 1 week between two adjacent experiments. Sixty-two channels EEG signals with the sampling frequency of 1,000 Hz were recorded. In addition, to save computing resources, the EEG signals were downsampled to 200 Hz. The artifact was also been removed. To evaluate the proposed model, the 15 trials EEG signals are divided into training data and testing data, where the training data contains first nine trials while the testing test contains the rest six trials from the same experiment. The EEG signals were filtered between 1 and 47 Hz by the fourth Butterworth bandpass filter, and a 1-s window with non-overlap was applied. To keep the number of samples in different categories consistent, we only selected the EEG signals of the last 2 min of each trial. The classification performance of the model is evaluated by accuracy as Equation (16),


(16) $$\begin{array}{l} Acc=\frac{TP+TN}{TP+FN+FP+TN} \end{array}$$

where TP (True Positives) indicates the number of samples that the model correctly predicts as positive categories, TN (True Negatives) represents the number of samples that the model correctly predicts as negative categories, FP (False Positives) indicates the number of samples in which the model incorrectly predicts negative categories as positive categories, and FN (False Negatives) represents the number of samples in which the model incorrectly predicts positive categories as negative categories.

The experimental environment was built on a Windows 10 PC with Core (TM) i7-10700 CPU, NVIDIA GeForce RTX 3080Ti, and the computing environment was pytorch 1.10.1.

### 3.2 Experimental results

To test the effectiveness of the four function connection features and the proposed method, we divide the input of the model into four modes:

- Only one type of feature: PLV, PCC, MI, and COH;
- The combination of two types of features: PLV+PCC, PLV+MI, PLV+COH, PCC+MI, PCC+COH, and MI+COH;
- The combination of three types of features: PLV+PCC+MI, PLV+PCC+COH, PLV+MI+COH, and PCC+MI+COH;
- The combination of all four types of features: PLV+PCC+MI+COH.

The experimental results are displayed in Table 3. As is shown in the table, the results for each feature mode is acceptable where the accuracies were higher than 83%. Besides, according to the Table 3, it can be also concluded that: (1) For the mode with only one feature, the PCC achieved highest performance (83.45%/7.20%) among the four functional connection features, while the PCC achieved highest performance (87.37%/5.5%), that is to say, MI is worse in accuracy and robustness in identifying the emotions of different subjects, while PCC is better than the other three features in these two aspects; (2) For the mode with the combination of two features, the PCC-COH achieved best accuracy, followed by the PLV-COH, PLV-PCC, PCC-MI, and PLV-MMI, and MI-COH achieved worst accuracy. An interesting phenomenon can be drawn that the average emotion recognition performances achieved by feature modes involving MI were always the worst; (3) For the mode with the combination of three features, the PLV-PCC-COH achieved the best mean accuracy, followed by the PCC-MI-COH, the PLV-MI-COH, and the PLV-PCC-MI. However, similar to the mode with two features, the feature modes involving MI still achieve poor performance; (4) For the mode with combination of all four features, the mean accuracy and standard deviation (std) for 15 subjects were 90.16% and 5.24%, respectively, where the accuracy was the second highest among all feature modes while the std was the lowest. It indicated that the four feature fusion modes can achieve more robust performance in emotion recognition tasks.

**Table 3:** The mean (standard deviation) accuracy of the proposed model with the four different feature modes (%).

**Feature**	**Accuracy**	**Feature**	**Accuracy**	**Feature**	**Accuracy**
PLV	86.27 (6.50)	PLV-MI	88.30 (5.81)	PLV-PCC-MI	89.46 (5.65)
PCC	87.37 (5.55)	PLV-COH	89.79 (6.01)	PLV-PCC-COH	**91.05 (5.54)**
MI	83.45 (7.20)	PCC-MI	88.52 (5.53)	PLV-MI-COH	89.50 (5.92)
COH	86.91 (6.64)	PCC-COH	89.86 (5.76)	PCC-MI-COH	89.97 (5.63)
PLV-PCC	89.59 (5.63)	MI-COH	88.02 (5.97)	PLV-PCC-MI-COH	90.16 (5.24)

The bold value represent the highest classification accuracy among all feature modes.

**Figure 4:**
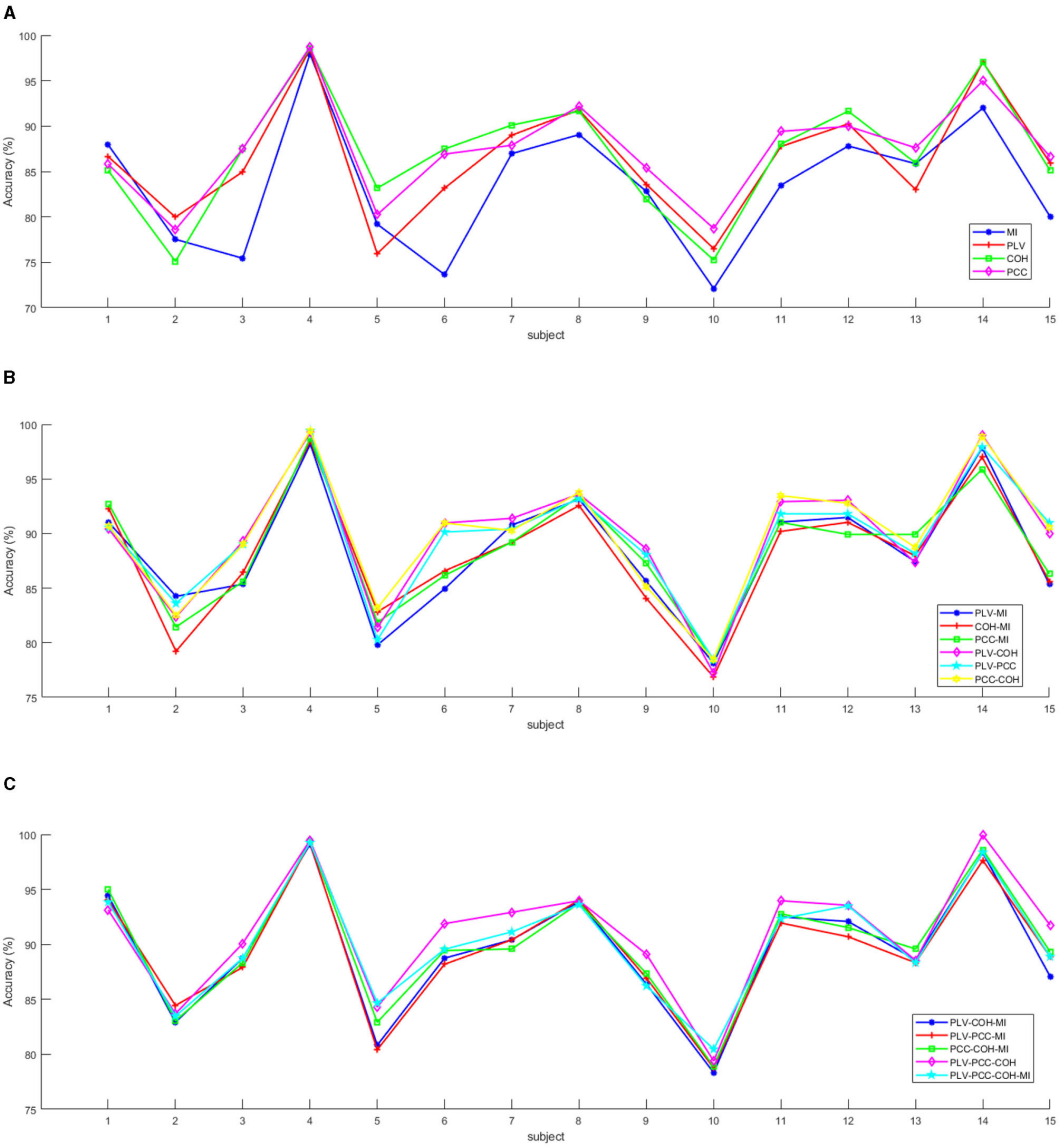
The mean accuracies for the 15 subjects with the different feature modes. **(A)** The mean accuracies for the 15 subjects with the mode of only one type of feature. **(B)** The mean accuracies for the 15 subjects with the mode of combination with two types of features. **(C)** The mean accuracies for the 15 subjects with the mode of combination with more than three types of features.

The detailed accuracy information for all the 15 subjects with all feature modes was shown in Figure 4. As is displayed in Figure 4A, the MI almost always achieved relatively low accuracies except for subject #1, while although the PCC did not always achieve the best accuracy, its value always kept stable and remained in the top two among the four features. Moreover, as is displayed in Figures 4B, C, for all 15 subjects, compared with a single feature mode, a combination of different number of features can effectively improve the performance of emotion recognition. In addition, for each subject, it can be found that compared with the difference in recognition performance between single feature modes, after the decision-making layer fusion of features, the performance difference of emotion classification models after different combinations of features is smaller. However, the PLV-PCC-COH almost always achieved best accuracies among all 15 subjects. In summary, combining Table 3 and Figure 4, the ability of PCC to measure brain functional connectivity (i.e., the interactive information between EEG electrodes) may be better than the other three functional connectivity features. In addition, for the fusion between features, PLV-PCC-COH is optimal in overall recognition performance, but the performance achieved when the four features are combined together is the most stable.

### 3.3 Influence of threshold

**Figure 5:**
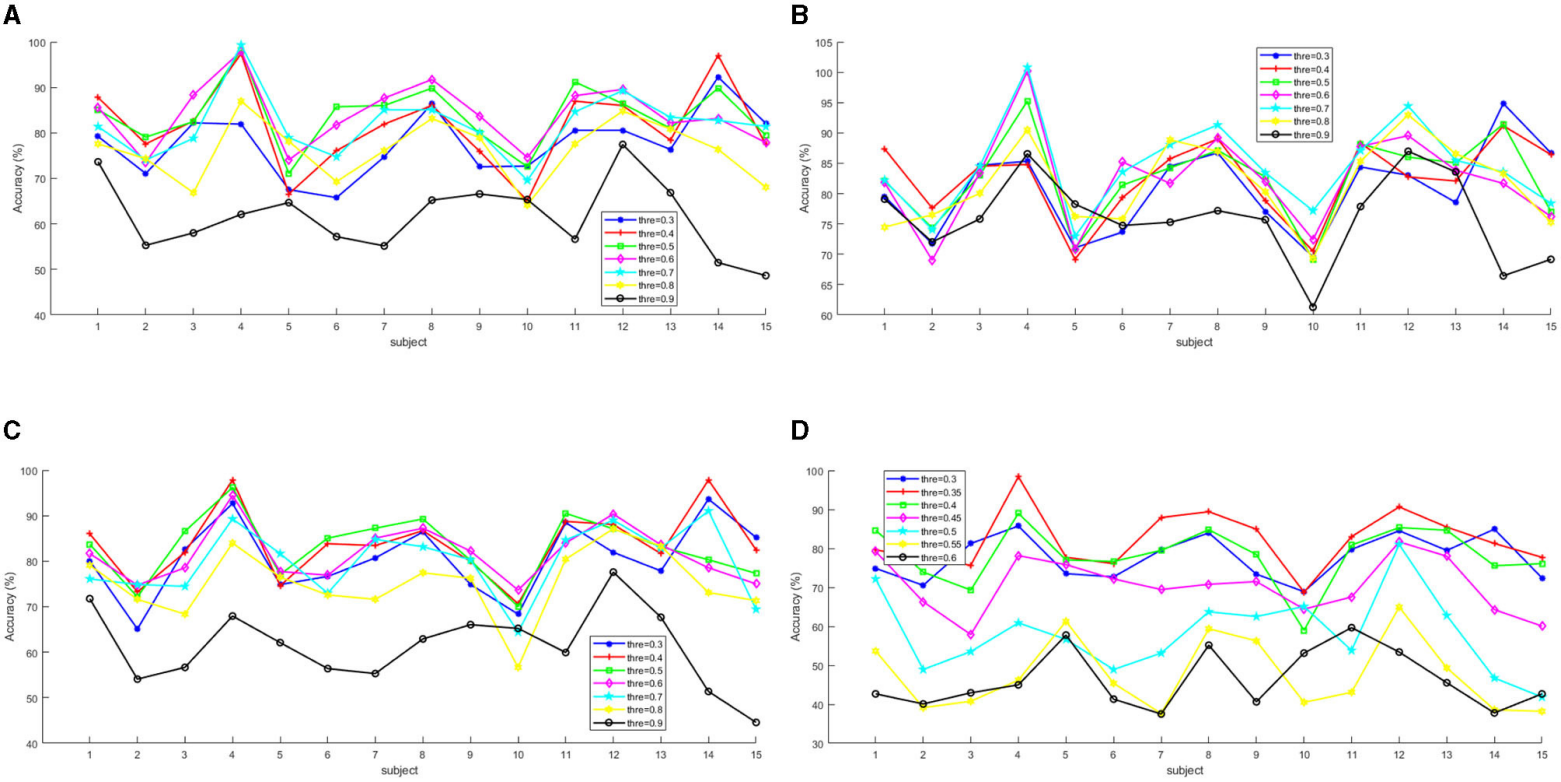
The accuracies of the 15 subjects for the four functional connected features with different threshold values. **(A)** PLV. **(B)** PCC. **(C)** COH. **(D)** MI.

In this part, we investigate the influence of the threshold to transform the function connection features into the undirected graph of EEG channels. Particularly, since the value range of PLV and COH is between [0, 1], and the value range of PCC is between [–1, 1], we chose the threshold value for the three features from the set {0.3,0.4,0.5,0.6,0.7,0.8,0.9}. Since the maximum value of MI is around 0.65, we chose the threshold value from the set {0.3,0.35,0.4,0.45,0.5,0.55,0.6}. The average and detailed results among 15 subjects are shown in Table 4 and Figure 5, respectively. As is shown in the table, we can conclude that for each functional connection feature, as the threshold increased, the feature's emotion recognition performance would increase. However, when it increased to a certain threshold, the feature's performance would begin to decline. More concretely, when the thresholds are 0.6, 0.7, 0.35, and 0.4 respectively, the classification accuracy of PLV, PCC, MI, and COH reaches the peak, respectively. It may be indicated that the optimal threshold values of the four features are in the range [0.5, 0.7], [0.6, 0.8], [0.3, 0.4], and [0.3, 0.5].

**Table 4:** The mean (standard deviation) accuracy of the proposed model for the four function connected features with different threshold values (%).

**Threshold**	**PLV**	**PCC**	**MI**	**COH**
0.30/0.30	77.74 (7.15)	80.75 (7.11)	77.71 (5.69)	80.63 (8.10)
0.40/0.35	81.53 (9.25)	82.51 (6.43)	**82.27 (7.41)**	**83.80 (7.76)**
0.50/0.40	83.86 (7.10)	82.54 (7.21)	78.32 (7.49)	83.05 (7.03)
0.60/0.45	**84.00 (6.98)**	82.31 (8.03)	70.48 (7.16)	81.58 (6.00)
0.70/0.50	81.90 (6.95)	**84.48 (7.43)**	58.12 (10.58)	79.94 (7.70)
0.80/0.55	76.22 (6.68)	81.50 (6.89)	47.63 (9.28)	75.29 (7.45)
0.90/0.60	61.63 (7.99)	76.00 (6.97)	46.35 (7.42)	61.31 (8.51)

The bold values in a single column represent the highest classification accuracy among all threshold values in each function connected feature.

In addition, according to Figure 5, it is obvious that for each subject, the optimal threshold is different, but the differences in the impact of different thresholds on emotion recognition performance were relatively stable. In other words, for PLV, the emotion recognition accuracies achieved when the thresholds are 0.5, 0.6, and 0.7, which were almost always at the forefront (shown in Figure 5A); for PCC, the emotion recognition accuracies achieved when the thresholds are 0.6, 0.7, and 0.8, which were almost always at the forefront (shown in Figure 5B); for COH, the emotion recognition accuracies achieved when the thresholds are 0.3, 0.4, and 0.5, which were almost always at the forefront (shown in Figure 5C); and for MI, the emotion recognition accuracies achieved when the thresholds are 0.3, 0.35, and 0.4, which were almost always at the forefront (shown in Figure 5D).

### 3.4 Comparison

Table 5 displayed the classification accuracies of parts of the state-of-the-art methods with the same training-test set partitioning strategy, i.e., 9 trials as training set and 6 trials as testing set for one experiment. As shown in the table, although the proposed model did not achieve the state-of-the-art performance, the method still outperforms most methods, which to some extent demonstrated the effectiveness of the proposed method. In addition, it can be obviously found that the DE feature is widely utilized in most methods to recognize emotions, while the functional connection features are employed relatively rarely. Therefore, the proposed method also demonstrates the effectiveness of functional connectivity features in identifying different emotions.

**Table 5:** The comparative results of the proposed model with other works (%).

**Method**	**Feature**	**Accuracy**
DBN (Zheng and Lu, 2015)	DE	86.06 (8.34)
GELM (Zheng et al., 2017)	DE	91.07 (7.54)
DGCNNN Song et al. (2018)	DE	90.40 (8.49)
STRNN (Zhang et al., 2018)	DE	89.50 (7.63)
GELM (Li et al., 2019)	DE, PLV-based ENPs	88.00 (7.00)
LSTM-ATDD (Du et al., 2020)	DE	91.08 (6.43)
RGNN (Zhong et al., 2020)	DE, GCN deep features	**94.24 (5.95)**
GNN (Lin et al., 2023)	DE, PLI	90.22 (3.67)
Ours model	PLV, PCC, COH, MI	91.05 (5.54)

The bold values in a single column represent the highest classification accuracy among all methods.

## 4 Conclusion and future works

In this study, we proposed a novel method which consists of the functional connection features, the Laplace matrix, and the SPDnet to perform the EEG-based emotion recognition, where the Laplace matrix was utilized to transform the functional connection features and the SPDnet was utilized to extract the deep spatial information from the transformed features. The proposed method achieved desirable performance on the SEED dataset for subject dependent cross-validation with the highest average accuracy of 91.05%/5.54% subject to the fusion of PLV, PCC, and COH. In addition, the experimental results showed that PCC has higher discriminability in identifying different emotions, while MI had the lowest discriminability. Although there are differences among the four functional connection features, the recognition performance were almost similar, especially for PLV, PCC, and COH, which may indicate that, when emotions are induced in subjects, the brain functional connections measured by different functions show a certain degree of stability. However, experimental results using different thresholds applied for the four functional connection features can also draw a similar conclusion. Furthermore, the experiment for different thresholds also indicated that for each subject, the optimal thresholds for the four functional connection features always kept relatively stable within a fixed interval.

However, the current study has certain limitations. We only tested the proposed model in the SEED dataset, which results in an inability to fully demonstrate the generalization performance of the model. Therefore, in future, we will test the model in more public datasets. In addition, this study briefly discusses the effectiveness of the proposed method. Of course, there is more information that can be mined, such as building a brain network and combining the proposed method with complex networks. Finally, we only considered the subject dependent cross-validation strategy, since there are differences between subjects that lead to inconsistent distribution of EEG data. Therefore, we will further combine the transfer learning to test the performance of the proposed model with the subject independent cross-validation strategy.

## Data availability statement

The original contributions presented in the study are included in the article/supplementary material, further inquiries can be directed to the corresponding author.

## Author contributions

MW: Conceptualization, Data curation, Formal analysis, Funding acquisition, Investigation, Methodology, Software, Writing – original draft, Writing – review & editing. RO: Conceptualization, Data curation, Formal analysis, Investigation, Methodology, Writing – original draft. CZ: Investigation, Software, Validation, Visualization, Writing – original draft. ZS: Investigation, Software, Validation, Visualization, Writing – original draft. PL: Conceptualization, Funding acquisition, Project administration, Supervision, Writing – review & editing. FL: Conceptualization, Supervision, Writing – review & editing.
